# Older Patients with Atopic Dermatitis Show More Pronounced Early Clinical Improvement with Tralokinumab: A Single-Center Retrospective Real-World Study

**DOI:** 10.3390/jcm15031117

**Published:** 2026-01-30

**Authors:** Emi Sato, Naoko Obonai, Monji Koga, Yoshitsugu Sibayama, Shinichi Imafuku

**Affiliations:** 1 Department of Dermatology, Fukuoka University Faculty of Medicine, Fukuoka 814-0180, Japan; n.obonai@fukuoka-u.ac.jp (N.O.); mob3mob3@yahoo.co.jp (M.K.); shibayoshi1978@hotmail.com (Y.S.); dermatologist@mac.com (S.I.); 2 Department of Dermatology, Fukuoka Sanno Hospital, Fukuoka 814-0001, Japan

**Keywords:** atopic dermatitis, older patients, tralokinumab

## Abstract

**Background/Objective**: Tralokinumab, a monoclonal antibody targeting interleukin-13, is an effective treatment for atopic dermatitis (AD). However, real-world data on age-related differences in clinical responses, particularly among older patients, remain limited. We compared early improvements in pruritus and skin lesions, as well as effectiveness, safety, and treatment persistence of tralokinumab, between older patients aged ≥ 70 and <70 years in real-world clinical practice. **Methods**: This single-center retrospective study included 43 patients with AD who initiated tralokinumab. Patients who discontinued treatment within 3 months, lacked a 3-month evaluation, or had a baseline Eczema Area and Severity Index (EASI) score < 16 were excluded, leaving 33 patients for effectiveness analyses. Patients were stratified by age (≥70 vs. <70 years). Outcomes at 3 months included pruritus severity assessed by the Peak Pruritus Numerical Rating Scale (PP-NRS), eczema severity assessed by the EASI, and response rates (PP-NRS4 and EASI75). Adverse events and reasons for treatment discontinuation were evaluated in all patients. **Results**: At 3 months, both age groups showed improvement in pruritus and skin lesions. Patients aged ≥ 70 years demonstrated more pronounced early improvement, with a median PP-NRS of 1 (interquartile range, 0–3), a PP-NRS4 response rate of 89.5%, and an EASI75 response rate of 84.2%. Treatment continuation rates did not differ significantly between age groups, indicating comparable tolerability. **Conclusions**: Tralokinumab was effective and well tolerated in both age groups, with older patients experiencing earlier and more pronounced clinical improvement. These findings suggest that tralokinumab may be effective in elderly patients with atopic dermatitis. These results may suggest tralokinumab as an effective therapy for elderly patients with atopic dermatitis. Validation using larger prospective studies is needed.

## 1. Introduction

In recent years, molecularly targeted therapies have substantially advanced the treatment of atopic dermatitis (AD) [1,2,3,4]. For patients with moderate-to-severe AD, multiple systemic options are now available, including Janus kinase inhibitors (JAKi) and biologic agents. JAKi rapidly alleviates pruritus by simultaneously inhibiting the interleukin (IL)-13 pathway, a central driver of AD pathophysiology, and the IL-31 signaling pathway, a major mediator of itch [5]. These agents can provide dramatic relief from pruritus within hours after oral administration. However, their broad inhibition of JAK–STAT signaling raises safety concerns, such as increased risk of skin infections and hematologic abnormalities, owing to impaired antiviral defense and altered hematopoiesis. Conversely, biologic agents target more specific immune pathways, such as the IL-4/IL-13 receptor, IL-13 itself, or the IL-31 receptor, resulting in a lower risk of systemic infections [6]. Nevertheless, except for super responders, biologics generally require longer periods to achieve maximal clinical efficacy [7]. In addition, unlike JAKi inhibitors, biologics cannot be dose-escalated, limiting their ability to rapidly control severe pruritus [8], extensive skin lesions, and quality of life (QOL) impairment in patients with very severe AD [9]. Furthermore, because biologics are administered by injection, they may be avoided by some patients with needle aversion. Epidemiologically, AD is no longer limited to childhood-onset disease associated with a classic atopic background [10]. The prevalence of adult- and late-onset AD, particularly in older adults, is increasing worldwide [11,12,13]. Older patients with AD experience severe pruritus and substantial QOL impairments, comparable to those observed in younger patients [14]. However, older individuals often have age-related immune dysfunction and frequently harbor multiple comorbidities, including diabetes mellitus, hypertension, dyslipidemia, cardiovascular and cerebrovascular diseases, and malignancies. Consequently, immunosuppressive therapies such as JAKi, cyclosporine, or systemic corticosteroids raise significant safety concerns in older patients with moderate-to-severe AD and are often discouraged in routine clinical practice [15]. Consequently, real-world studies indicate that biologic therapies—most commonly dupilumab, an anti-IL-4/IL-13 receptor antibody—are preferentially selected as systemic treatments for older patients with moderate-to-severe AD [14]. However, diagnosing and treating older patients with AD requires caution, as some patients may have early-stage cutaneous T-cell lymphoma (CTCL), particularly the patch stage of mycosis fungoides [15,16]. A large-scale TriNetX database analysis in the United States demonstrated an increased incidence of CTCL in patients with AD treated with dupilumab compared with untreated patients (relative risk [RR]: 4.59; 95% confidence interval [CI]: 2.459–8.567; *p* < 0.0001) [17]. Subgroup analysis further revealed that 54.5% of CTCL cases occurring after dupilumab initiation were observed in patients aged ≥ 60 years [17]. Consistently, multiple case reports and series have documented CTCL unmasking or progression following dupilumab treatment [18,19,20], prompting the Japanese Dermatological Association to recommend careful monitoring of skin lesions during treatment [21]. Mechanistically, previous basic studies have demonstrated strong expression of the IL-13 receptor α2 (IL-13Rα2) in CD4-positive lymphocytes derived from patients with CTCL [22]. IL-13 signaling inhibition or exogenous IL-13Rα2 modulation suppresses the proliferation of these malignant T cells, suggesting that enhanced IL-13Rα2 signaling may promote CTCL cell growth [22]. Moreover, recent studies have implicated IL-13Rα2 expression in the progression, metastasis, recurrence, and prognosis of several malignancies, including colorectal and breast cancers, prompting the development of immunotherapies targeting this pathway [23,24]. Currently, three IL-13–related biologics are approved for AD treatment—dupilumab, tralokinumab, and lebrikizumab—among which tralokinumab is the only agent that directly neutralizes IL-13 and inhibits IL-13Rα2 signaling [25]. This unique mechanism may be particularly advantageous when considering safety in older patients with multiple comorbidities. In secondary analyses of phase III trials focusing on patients aged ≥ 65 years, tralokinumab showed no increase in notable adverse events [26]. In addition, a multicenter retrospective non-interventional study conducted in Spain involving patients aged ≥ 65 years (N = 24, mean age 75.3 years) reported favorable effectiveness, with Eczema Area and Severity Index (EASI)75 and EASI90 response rates of 69.6% and 47.8% at Week 16, respectively, suggesting that tralokinumab may be especially effective in very older patients [27].

## 2. Materials and Methods

### 2.1. Study Design and Patients

**Figure 1:**
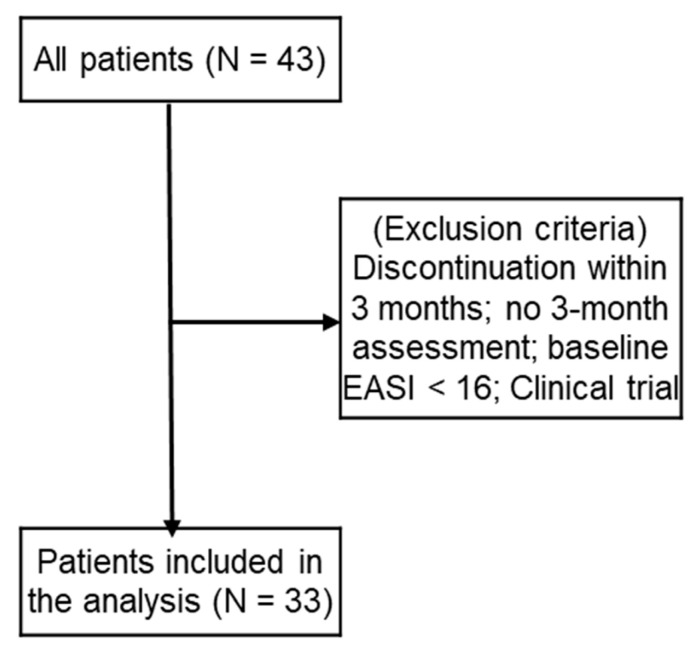
Patient selection flowchart. From the 43 patients who started with tralokinumab, 33 patients with 3-month available assessments were included in efficacy analysis. Safety outcomes, reasons for treatment discontinuation, and subsequent treatments were evaluated among all 43 subjects who received at least one dose of tralokinumab. EASI, Eczema Area and Severity Index.

This retrospective, single-center observational study was conducted at the Department of Dermatology, Fukuoka University Hospital, Japan. Medical records of patients diagnosed with AD who initiated tralokinumab treatment were collected. A total of 43 patients who received at least one dose of tralokinumab were initially screened. Patients were excluded from the effectiveness analysis if they met any of the following criteria: (1) tralokinumab discontinuation within 3 months after initiation, (2) absence of clinical evaluation at 3 months, (3) baseline EASI score < 16, or (4) treatment with tralokinumab as part of a clinical trial. After applying these criteria, 33 patients were included in the effectiveness analysis (Figure 1). Analyses of adverse events, reasons for treatment discontinuation, and subsequent treatments were conducted in all 43 patients who received tralokinumab. Patients were stratified according to age at treatment initiation into two groups: patients aged ≥ 70 and <70 years. Differential diagnoses that are more common in elderly patients, such as CTCL, were routinely considered. Diagnostic evaluation generally included skin biopsy with histopathological examination, as well as peripheral blood testing, including complete blood counts with differential and measurement of soluble IL-2 receptor levels, particularly in patients with atypical clinical features, late-onset disease, or an inadequate response to treatment.

### 2.2. Treatment Regimen

Tralokinumab was administered in accordance with the approved prescribing information in Japan, with a 600 mg loading dose followed by subcutaneous injections of 300 mg every 2 weeks. Concomitant topical therapy, including topical corticosteroids, was continued in all patients as part of routine clinical care. No restrictions were placed on emollient use.

### 2.3. Laboratory Assessments

Routine laboratory data obtained in clinical practice were reviewed, including serum thymus and activation-regulated chemokine (TARC), total immunoglobulin E (IgE), and peripheral blood eosinophil counts. Laboratory testing was performed at the discretion of the treating physician according to standard clinical practice.

### 2.4. Clinical Outcome Measures

Disease severity was evaluated using the EASI and Investigator’s Global Assessment (IGA) validated composite measures of AD severity. Pruritus severity was assessed using the Peak Pruritus Numerical Rating Scale (PP-NRS). Clinical effectiveness at 3 months was assessed using both continuous and categorical endpoints. Categorical response outcomes were defined as: EASI75, representing a ≥75% reduction from baseline EASI score; PP-NRS4, representing a ≥4-point reduction from baseline PP-NRS score; and IGA 0/1, defined as an IGA score of clear (0) or almost clear (1).

### 2.5. Safety and Discontinuation Assessment

Adverse events occurring during tralokinumab treatment were identified through review of medical records and recorded regardless of the suspected causal relationship to tralokinumab. Adverse events were summarized descriptively. Reasons for treatment discontinuation were categorized based on the primary clinical judgment of the treating physician when multiple contributing factors were present. Each patient was assigned a single primary reason for discontinuation.

### 2.6. Treatment Persistence Analysis

Treatment persistence was defined as the duration from tralokinumab initiation to treatment discontinuation for any reason. Drug survival was evaluated using the Kaplan–Meier method, and differences between age groups were assessed using the log-rank (Mantel–Cox) test. Median treatment duration was estimated for each age group.

### 2.7. Statistical Analysis

Continuous variables are presented as medians with interquartile ranges (Q1–Q3), and categorical variables are presented as counts and percentages. Longitudinal changes in EASI and PP-NRS between age groups were analyzed using two-way analysis of variance (ANOVA) with Bonferroni post hoc correction. Between-group comparisons of categorical response rates (EASI75) were conducted using Fisher’s exact test. Statistical significance was defined as a two-sided *p*-value < 0.05. All statistical analyses were performed using GraphPad Prism (version 5; GraphPad Software, San Diego, CA, USA). Multivariate logistic regression analyses were performed using JMP software (version 18.2.0; SAS Institute Inc., Cary, NC, USA). Forest plots were generated using EZR (Jichi Medical University, Tochigi, Japan), based on R version 4.3.1.

## 3. Results

### 3.1. Patient Background and Characteristics

Forty-three patients with AD treated with tralokinumab were screened. Patients who discontinued treatment within 3 months, lacked a 3-month clinical assessment, had baseline EASI scores < 16, or participated in a clinical trial were excluded. Effectiveness outcomes were assessed in these 33 patients with 3-month available assessments, whereas safety outcomes were assessed in all 43 patients who received at least 1 dose of tralokinumab unless otherwise specified. Adverse events, reasons for discontinuation and subsequent treatment were analyzed for all 43 patients receiving tralokinumab (Figure 1). Among the 10 patients excluded from the efficacy analysis, the reasons for exclusion were discontinuation before 3 months due to insufficient effectiveness (≥70 years: n = 2; <70 years: n = 1), discontinuation before 3 months due to social reasons (≥70 years: n = 1; <70 years: n = 0), discontinuation before 3 months due to early clinical improvement (≥70 years: n = 1; <70 years: n = 0), discontinuation before 3 months due to adverse events (≥70 years: n = 0; <70 years: n = 1), less than 3 months after treatment initiation (≥70 years: n = 0; <70 years: n = 1), and baseline EASI < 16 (≥70 years: n = 2; <70 years: n = 1).

Table 1 summarizes patient baseline characteristics. The group aged ≥ 70 years old comprised 19 cases (2 women), whereas the group < 70 years old comprised 14 cases (6 women). The proportion of men was significantly higher in the group aged ≥ 70 years (*p* = 0.047). The median disease duration [Q1–Q3] was 4 years (1–20) in the ≥70 years group, significantly shorter than the 19 years (5–45.25) in the <70 years group (*p* = 0.017). No significant between-group differences were observed regarding body weight, atopic predisposition, or proportion receiving systemic therapy. Conversely, the proportion of bio-naïve patients was significantly higher in the ≥70 years group (18 vs. 8 patients, *p* = 0.026). Disease severity at initiation was higher in the ≥70 years group, with a median PP-NRS [Q1–Q3] of 8 (6–10) compared with 6.5 (4.5–7) in the <70 years group (*p* = 0.005). Similarly, the median EASI score was significantly higher in the ≥70 years group (23.8 [19.6–33.2]) than in the <70 years group (18.4 [16–20.25]; *p* = 0.0011), reflecting the clinical reality that in older patients, immunosuppressive therapies such as JAKi or cyclosporine are less likely to be selected owing to concerns about complications, resulting in higher disease activity at the time of tralokinumab initiation. Among hematologic parameters, no significant between-group differences were observed in eosinophil count or serum IgE levels. However, serum TARC levels were significantly higher in the ≥70 years group (3390 vs. 734 pg/mL, *p* = 0.0027), suggesting stronger disease activity in the group. Our facility tended to select nemolizumab for intrinsic AD cases presenting with severe pruritus as the primary complaint but lacking atopic predisposition [28]. Consequently, patients without atopic predisposition were relatively few in both groups.

### 3.2. Efficacy of Tralokinumab

**Figure 2:**
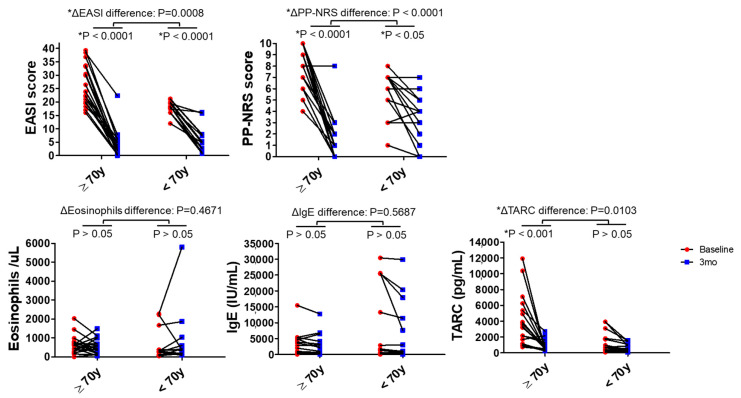
Clinical outcomes and biomarker changes following tralokinumab by age group. Changes in the Eczema Area and Severity Index (EASI), Peak Pruritus Numerical Rating Scale (PP-NRS), eosinophil counts, serum Immunoglobulin E (IgE) levels, and serum thymus and activation-regulated chemokine (TARC) levels from baseline to 3 months after tralokinumab initiation are shown for patients aged ≥ 70 and <70 years. Each symbol represents an individual patient, with red circles indicating baseline values and blue squares indicating values at 3 months. Statistical analyses were performed using two-way analysis of variance with Bonferroni post hoc tests. Statistically significant: *p* < 0.05.

The changes in clinical symptoms and biomarkers following tralokinumab treatment were compared between groups, as shown in Figure 2 and Table 2. From baseline to 3 months, both groups showed significant improvements in EASI and PP-NRS scores (Figure 2).

Two-way ANOVA demonstrated significant between-group differences in the changes in EASI (ΔEASI, *p* = 0.0008) and PP-NRS (ΔPP-NRS, *p* < 0.0001), indicating greater improvement in patients aged ≥ 70 years. In within-group analyses, EASI (both *p* < 0.0001) and PP-NRS (*p* < 0.0001, patients aged ≥ 70 years; *p* < 0.05, patients aged < 70 years) scores significantly decreased in both groups.

As shown in Table 2, analysis of clinical response rates at 3 months revealed that the median PP-NRS [Q1–Q3] was 1 (0–3) in patients aged ≥ 70 years, significantly lower than 4 (1.75–5) in patients aged < 70 years (*p* = 0.0103). The PP-NRS4 response rate was significantly higher in patients aged ≥ 70 years (17/19, 89.5%) than in those aged < 70 years (4/14, 28.6%; *p* = 0.0006). No significant between-group difference was observed in the PP-NRS0/1 response rate (*p* = 0.0733). Regarding skin severity, the EASI75 response rate at 3 months was significantly higher in patients aged ≥ 70 years (16/19, 84.2%) than in patients aged < 70 years (6/14, 42.9%; *p* = 0.024). Conversely, no significant between-group difference was observed in the EASI90 response rate (*p* = 0.1604). Regarding inflammatory biomarkers, as shown in Figure 2, eosinophil counts and serum IgE levels exhibited no significant changes before and after treatment in either group, and no significant between-group differences in their changes were observed. Conversely, serum TARC levels significantly decreased in patients aged ≥ 70 years (*p* < 0.001), and the magnitude of change differed significantly between groups (ΔTARC, *p* = 0.0103). No significant change in TARC levels was observed in patients aged < 70 years.

**Figure 3:**
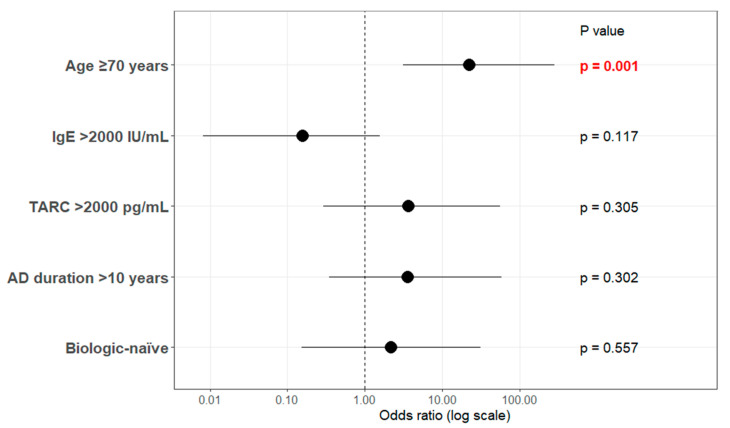
Forest plot of multivariate logistic regression analysis using simultaneous achievement of EASI75 and PP-NRS4 at 3 Months as the outcome variable. Multivariate logistic regression analysis was performed using stepwise variable selection. Candidate variables included age ≥ 70 years, baseline IgE > 2000 IU/mL, baseline TARC > 2000 pg/mL, disease duration > 10 years, and bio-naïve. Odds ratios are presented on a logarithmic scale, with horizontal lines indicating 95% CI. Model validity was assessed using the likelihood ratio test (χ^2^ = 17.49, *p* = 0.0037), the lack of fit test (LOF, *p* = 0.136), and pseudo R^2^ (R^2^_U = 0.383). The final model was estimated based on 33 observations. Statistical significance was assessed using Wald and likelihood ratio tests. Statistical significance: *p* < 0.05. IgE, immunoglobulin E; TARC, thymus and activation-regulated chemokines; AD, atopic dermatitis; CI, confidence interval; EASI, Eczema Area and Severity Index; PP-NRS, Peak Pruritus Numerical Rating Scale. Red-colored *p*-values indicate statistically significant associations (*p* < 0.05).

Finally, multiple logistic regression analysis using a stepwise variable selection procedure was performed to identify predictors of simultaneous achievement of PP-NRS4 and EASI75 at 3 months (Figure 3). Candidate variables selected include age ≥ 70 years, high baseline IgE (>2000 IU/mL), high baseline TARC (>2000 pg/mL), disease duration > 10 years, and biologic-naïve status. The analysis identified age ≥ 70 years as the only independent predictor of achieving both PP-NRS4 and EASI75 (odds ratio: 22.1, 95% CI: 3.10–280.63; *p* = 0.0014). The other variables were not significant predictors. The overall model was significant (likelihood ratio test, *p* = 0.0037), and model fit was acceptable (LOF test, *p* = 0.136).

### 3.3. Treatment Persistence and Reasons for Discontinuation

**Figure 4:**
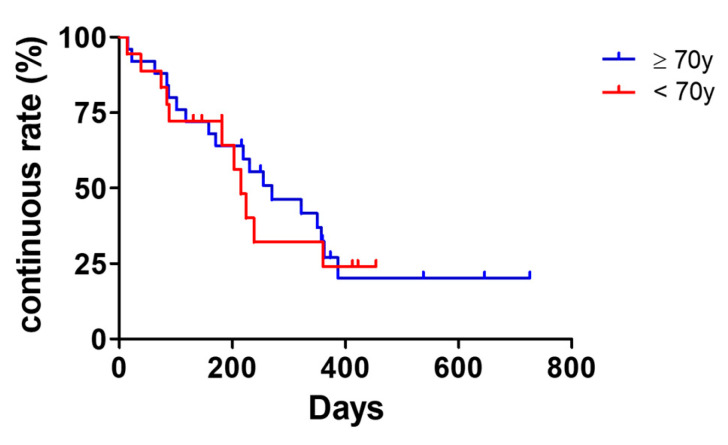
Tralokinumab treatment continuation by age group. Kaplan–Meier curves depict treatment continuation over time in patients aged ≥ 70 years and those aged < 70 years. Treatment persistence was compared between groups using the log-rank (Mantel–Cox) test. Median treatment duration for each group is indicated in the figure. A two-sided *p*-value < 0.05 was considered statistically significant.

Tralokinumab treatment persistence stratified by age group is shown in Figure 4. Kaplan–Meier survival analysis demonstrated no significant difference in treatment continuation between patients aged ≥ 70 and <70 years (log-rank [Mantel–Cox] test, *p* = 0.1543). The median treatment durations were 270 and 215 days in patients aged > 70 and <70 years, respectively.

Reasons for treatment discontinuation are summarized in Table 3. Overall discontinuation rates were comparable between groups (*p* = 0.5205). Notably, discontinuation owing to achievement of remission accounted for a substantial proportion of treatment discontinuations in both groups and was one of the most frequent reasons, particularly in patients aged ≥ 70 years (≥70 years: 4/25 patients, 16.0%; <70 years: 2/18 patients, 11.1%). In addition to remission, some patients discontinued treatment because of reduced or insufficient effectiveness. Pruritus and nodular prurigo unrelated to eczematous lesions led to treatment discontinuation in 3 of 25 patients (12.0%) in the ≥70-year group. In the <70-year group, one patient experienced pruritus, with the reason for discontinuation being marked eosinophilia. Treatment discontinuation owing to adverse events was infrequent in both age groups.

### 3.4. Safety Profile and Subsequent Treatment

Adverse events observed during tralokinumab treatment are summarized in Table 4. The overall incidence of adverse events was comparable between groups (*p* = 0.7548). The most frequently observed adverse event in the ≥70-year group was pruritus or prurigo nodularis, occurring in 5/25 patients (20.0%), whereas only one patient (5.6%) in the <70-year group experienced this event. Other adverse events, including eye itching or conjunctivitis, joint pain, drug eruption, and injection-site pain leading to treatment discontinuation, were infrequent and occurred at similarly low rates in both age groups.

Subsequent treatments after tralokinumab discontinuation are summarized in Table 5. Among patients who discontinued tralokinumab, the choice of subsequent therapy did not differ significantly between groups.

In both groups, switching to another biologic agent was the most common strategy, with lebrikizumab and dupilumab used in a subset of patients. Notably, nemolizumab was administered in four patients who developed pruritus or prurigo nodularis after tralokinumab treatment, resulting in marked improvement of itch symptoms in all cases. This finding suggests that IL-31 pathway inhibition may be an effective option for managing refractory pruritus or prurigo nodularis emerging during IL-13–targeted therapy.

**Figure 5:**
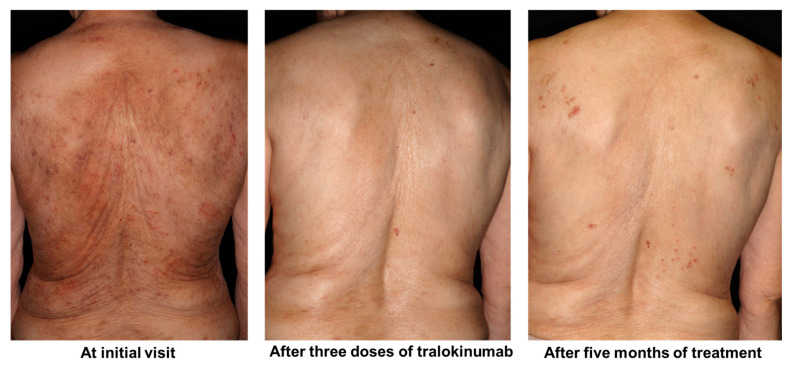
Clinical course of an older patient treated with tralokinumab. Clinical photographs of a 78-year-old woman with AD. At the initial visit (**left**), after three doses of tralokinumab (**center**), and 5 months after treatment initiation (**right**). Eczematous skin lesions improved rapidly and were well controlled after treatment initiation. However, despite sustained improvement in dermatitis, new-onset pruritus and nodular prurigo developed during the course of treatment. AD, atopic dermatitis.

A representative case is shown in Figure 5. A 78-year-old woman with AD showed rapid clinical improvement after initiation of tralokinumab therapy. However, despite sustained improvement in eczematous skin lesions, she developed new-onset pruritus and nodular prurigo that differed from her pretreatment symptoms approximately 5 months after treatment initiation.

Although she had a family history of AD and an atopic predisposition, her baseline serum IgE level before tralokinumab treatment was relatively low (135 IU/mL). Therefore, nemolizumab, an anti–IL-31 receptor A antibody, was initiated.

Pruritus resolved after a single dose of nemolizumab, and the treatment has been continued with sustained symptom control.

## 4. Discussion

This single-center retrospective study compared the real-world efficacy, safety, treatment persistence, and reasons for discontinuation of tralokinumab in patients with AD stratified by age (≥70 vs. <70 years). In this study, patients aged 70 years or older tended to show more pronounced early improvement in skin lesions and pruritus compared to younger patients. While this observation is noteworthy, it should be interpreted cautiously given the exploratory nature of the analysis. In the present study, significant improvements in EASI and PP-NRS scores were observed in both age groups at 3 months after treatment initiation; however, the magnitude of improvement (ΔEASI and ΔPP-NRS) was significantly greater in the ≥70-year group (Figure 2). In addition, the achievement rates of PP-NRS4 and EASI75, as well as the concurrent achievement of both endpoints, were significantly higher among patients aged ≥ 70 years (Table 2). In exploratory multivariate logistic regression analysis, age 70 years or older was the only variable associated with achieving both PP-NRS4 and EASI75. However, given the limited sample size and wide confidence intervals, this result should be interpreted with caution. (Figure 3). Combining these findings suggests that some AD patients may exhibit increased early responsiveness to tralokinumab. However, this hypothesis requires verification through larger prospective studies. Notably, patients aged ≥ 70 years had higher baseline EASI, PP-NRS, and TARC levels than those aged < 70 years, indicating greater disease activity at treatment initiation. This difference in baseline likely indicates real-world treatment selection, where systemic immunosuppressive agents such as JAKi or cyclosporine are often avoided in the elderly population for safety concerns and result in preferential use of biologics (e.g., tralokinumab) even in cases of severe disease. Despite this, treatment responsiveness was superior in the older patient group. This observation cannot be adequately explained by the assumption that patients improved simply because their disease was milder at baseline. Rather, they may also reflect variation of actual aging disease traits and treatment choice behaviours among actual aging population in human care, for example, IL-13–dominant inflammatory features detected in a substantial subset of aging patients with AD.

Previous real-world studies have generally reported greater early responsiveness to tralokinumab in younger patients. For example, a prospective observational study by Alegre-Bailo et al. demonstrated that super-responders were more frequently younger individuals [29], whereas a large prospective study from Japan by Hagino et al. showed that early responders were more likely to be younger, women, and have a lower body mass index [30]. Conversely, a retrospective multicenter study limited to older patients (≥65 years) by Melgosa Ramos et al. reported sustained efficacy and a favorable safety profile of tralokinumab in older adults [27]. In addition, a recent real-world study by Lauletta et al. demonstrated significant clinical improvement and acceptable safety of tralokinumab in elderly patients aged ≥ 60 years in routine clinical practice [31]. While the overall direction of our findings is consistent with these reports, several important differences should be noted. While the studies by Melgosa Ramos et al. and Lauletta et al. primarily focused on overall efficacy and safety through long-term follow-up in elderly patients (aged ≥ 60 or 65 years), our study targeted very older population (aged ≥ 70 years) and specifically evaluated early treatment response at 3 months. Furthermore, our analysis directly compared outcomes between elderly and younger patients within the same real-world clinical setting. It should also be noted that, although the prevalence of intrinsic AD is thought to be relatively high in the elderly, the number of intrinsic AD patients included in this tralokinumab treatment cohort was small. At our institution, based on the mechanism of action and clinical experience, nemolizumab is preferred for intrinsic AD patients without concomitant atopic disease and without markedly elevated serum IgE levels [28]. Therefore, the tralokinumab treatment group in this study primarily consisted of patients with extrinsic or IgE-related disease. However, in this population, we complement existing multicenter real-world data by providing additional insights, particularly regarding patterns of early clinical response associated with aging, in the elderly population (aged ≥ 70 years), which remains underrepresented in both clinical trials and real-world studies. These observations complement existing real-world data but remain at an exploratory stage.

These results suggest that the relationship between age and treatment response is not linear and raise the possibility that IL-13–dependent inflammation is particularly prominent in specific disease phenotypes, such as late- or older-onset AD. Previous studies have shown that the proportion of male patients increases with age [11] and that intrinsic AD becomes more prevalent in older populations, often accompanied by relatively low serum IgE levels [32]. Nevertheless, inflammatory markers such as TARC frequently remain elevated, suggesting the persistence of IgE-independent type 2 inflammation [33]. In our study, significantly higher baseline TARC levels in the ≥70-year group further support the involvement of IL-13–driven inflammation in older patients with AD. Moreover, exploratory analyses revealed a trend toward an inverse correlation between the magnitude of pruritus improvement and age at disease onset, indicating that patients with later-onset AD may experience greater pruritus relief. Together, these findings suggest that older individuals with AD may represent a disease state in which both skin inflammation and pruritus are particularly responsive to IL-13 inhibition. Another important observation of this study is that several older patients developed new-onset pruritus or prurigo nodularis without accompanying eczematous lesions after initial improvement of both skin lesions and pruritus with tralokinumab (Table 4). In a representative case (Figure 4), pruritus and prurigo nodularis emerged 5 months after treatment initiation despite sustained dermatitis improvement, suggesting that although IL-13 inhibition may initially improve both skin inflammation and pruritus in older individuals with AD, pruritus mediated predominantly through IL-31–dependent pathways may subsequently become relatively unmasked. Further, in this study, nemolizumab (an anti–IL-31 receptor A antibody) was introduced in patients who developed pruritus or prurigo nodularis after tralokinumab treatment, resulting in pruritus improvement in all cases. These observations provide clinically meaningful evidence that pruritus in older patients with AD represents a multilayered pathophysiological process that cannot be explained by a single inflammatory pathway.

Regarding treatment persistence, no significant difference was observed between patients aged ≥ 70 and <70 years. Notably, “achievement of remission” was a relatively frequent reason for treatment discontinuation in both age groups. This finding reflects real-world clinical decision-making in which treatment is temporarily discontinued once sufficient symptom control has been achieved and indirectly supports the high clinical effectiveness of tralokinumab.

This study has some limitations. First, it was a single-center retrospective study with a limited sample size. Second, efficacy analyses were limited to patients with available 3-month assessments, opening up the possibility of survivorship bias, as patients who discontinued treatment early were excluded. Though the reasons for exclusion in this study were well-documented, this design could have affected the effectiveness estimates recorded. Third, there were differences in baseline characteristics between age groups, such as disease severity, biologic-naïve status, and disease duration. In the case of elderly patients, these imbalances reflect real-life patient choice, especially for those who are immune-deficient and whom systemic immunosuppressive agents such as JAKi or cyclosporine are often refrained from (for reasons of safety). But they serve as confounding factors even here and are unable to infer causally between the age differences in treatment response. Fourth, the number of patients with intrinsic atopic dermatitis was small, reflecting institutional treatment preferences, which may limit the applicability of the results to this subgroup. Further, while relevant differential diagnoses that are more common in older populations (e.g., CTCL) were carefully considered and excluded on clinical, histopathological, and laboratory examination, subclinical disease cannot be definitively excluded. Finally, the small observation time limits the interpretation about long-term efficacy and safety.

Overall, this study should be considered hypothesis-generating, and a larger prospective multicenter cohort study is needed to verify the observed age-related differences in early response to tralokinumab.

## Figures and Tables

**Table 1:** Background and characteristics of patients categorized as ≥70 and <70 years old.

Baseline	≥70 y	<70 y	*p*-Value
Total, N (Female, N)	19 (2)	14 (6)	0.0473 †
Age, y, median (Q1, Q3)	82 (74, 86)	50.5 (27.75, 60)	-
Disease duration, y, median (Q1, Q3)	4 (1, 20)	19 (5, 45.25)	0.0173 ¶
Body weight, kg, median (Q1, Q3)	59.6 (50.75, 65.6)	58 (52.88, 85)	0.4358 ¶
w/o atopic predisposition, N	3	2	1.0000 †
Systemic therapy-naïve, N	10	5	0.4824 †
Bio-naïve, N	18	8	0.0260 †
PP-NRS, median (Q1, Q3)	8 (6, 10)	6.5 (4.5, 7)	0.0050 ¶
EASI, median (Q1, Q3)	23.8 (19.6, 33.2)	18.4 (16, 20.25)	0.0011 ¶
IGA, median (Q1, Q3)	3 (3, 4)	3 (3, 3)	0.1638 ¶
Eosinophil counts/μL, median (Q1, Q3)	610 (276, 740)	280 (124.3, 705.8)	0.1502 ¶
IgE, IU/mL, median (Q1, Q3)	964 (167, 4608)	1620 (335.5, 25,480)	0.4125 ¶
TARC, pg/mL, median (Q1, Q3)	3390 (1156, 5356)	734 (335.3, 2140)	0.0027 ¶

† Fisher’s exact test, ¶ Mann–Whitney U test.

**Table 2:** Clinical outcomes and biomarker levels at 3 months by age group.

Three Months After Treatment	≥70 y	<70 y	*p*-Value
Total, N	19	14	
PP-NRS, median (Q1, Q3)	1 (0, 3)	4 (1.75, 5)	0.0103 ¶
PP-NRS4, N	17	4	0.0006 †
PP-NRS0/1, N	11	3	0.0733 †
EASI, median (Q1, Q3)	3 (1.8, 5.9)	5 (2.25, 8)	0.1500 ¶
EASI 75, N	16	6	0.0240 †
EASI 90, N	9	3	0.1604 †
IGA, median (Q1, Q3)	1 (1, 2)	2 (1, 2)	0.2084 ¶
IGA0/1, N	11	6	0.4905 †
Both PP-NRS4 and EASI75 achieved, N	14	2	0.0013 †
Both PP-NRS4 and IGA0/1 achieved, N	11	1	0.0036 †
Eosinophil counts/μL, median (Q1, Q3)	460 (211, 696)	364 (164, 715)	0.5004 ¶
IgE, IU/mL, median (Q1, Q3)	687 (124, 3287)	986 (334.3, 13,050)	0.4125 ¶
TARC, pg/mL, median (Q1, Q3)	531 (402, 1070)	517.5 (318.8, 1185)	0.5974 ¶

† Fisher’s exact test, ¶ Mann-Whitney U test.

**Table 3:** Reasons for tralokinumab discontinuation by age group.

Reasons for Discontinuation	≥70 y	<70 y	*p*-Value †
Total patients, N	25	18	
Total discontinuation, N (%)	18 (72)	11 (61.1)	0.5205
Effect reduction, N (%)	4 (16)	3 (16.7)	1.0000
Insufficient effect, N (%)	3 (12)	4 (22.2)	0.4274
Remission, N (%)	4 (16)	2 (11.1)	1.0000
Pruritus/prurigo nodularis, N (%)	3 (12)	0 (0)	0.2525
Social reason, N (%)	2 (8)	0 (0)	0.5017
Drug eruption *, N (%)	1 (4)	1 (5.6)	1.0000
Grade 3 eosinophilia, N (%)	0 (0)	1 (5.6)	0.4186
Pain at the injection site, N (%)	1 (4)	0 (0)	1.0000

* The causal relationship was unclear or unlikely. † Fisher’s exact test.

**Table 4:** Adverse events occurring after tralokinumab by age group.

Adverse Events	≥70 y	<70 y	*p*-Value †
Total patients, N	25	18	
Total adverse events, N (%)	10 (40)	6 (33.3)	0.7548
Pruritus/prurigo nodularis, N (%)	5 (20)	1 (5.6)	0.3747
Eye itching/conjunctivitis, N (%)	2 (8)	2 (11.1)	1.0000
Joint pain, N (%)	1 (4)	1 (5.6)	1.0000
Drug eruption *, N (%)	1 (4)	1 (5.6)	1.0000
Grade 3 eosinophilia, N (%)	0 (0)	1 (5.6)	0.4186
Pain at the injection site leading to discontinuation, N (%)	1 (4)	0 (0)	1.0000

* The causal relationship was unclear or unlikely. † Fisher’s exact test.

**Table 5:** Treatment following tralokinumab by age group.

Next Treatment	≥70 y	<70 y	*p*-Value †
Total discontinuation, N	18	11	
Lebrikizumab (IL-13 ab), N (%)	4 (22.2)	6 (54.5)	0.1142
Topical treatment only, N (%)	6 (33.3)	2 (18.2)	0.6706
Dupilumab (IL-4/13R ab), N (%)	3 (16.7)	1 (9)	1.0000
Nemolizumab (IL-31R ab), N (%)	3 (16.7)	1 (9)	1.0000
Abrocitinib (JAK1 inhibitor), N (%)	1 (5.6)	0 (0)	1.0000
Oral predonisolone, N (%)	1 (5.6)	1 (9)	1.0000

† Fisher’s exact test.

## Data Availability

The datasets generated and analyzed during the current study are not publicly available due to institutional and ethical restrictions but are available from the corresponding author upon reasonable request and with permission from the institutional review board.

## Abbreviations

The following abbreviations are used in this manuscript:

AD	Atopic dermatitis
EASI	Eczema Area and Severity Index
IGA	Investigator’s Global Assessment
PP-NRS	Peak Pruritus Numerical Rating Scale
JAKi	Janus kinase inhibitors
QOL	Quality of life
CTCL	Cutaneous T-cell lymphoma
IL	Interleukin
IL-13Rα2	IL-13 receptor α2
TARC	thymus and activation-regulated chemokine
IgE	Immunoglobulin E
